# Diacylglycerol Kinase-ε: Properties and Biological Roles

**DOI:** 10.3389/fcell.2016.00112

**Published:** 2016-10-18

**Authors:** Richard M. Epand, Vincent So, William Jennings, Bijendra Khadka, Radhey S. Gupta, Mathieu Lemaire

**Affiliations:** ^1^Department of Biochemistry and Biomedical Sciences, McMaster University Health Sciences CentreHamilton, ON, Canada; ^2^Nephrology Division and Cell Biology Program, Hospital for Sick ChildrenToronto, ON, Canada; ^3^Department of Biochemistry, University of TorontoToronto, ON, Canada; ^4^Institute of Medicine, University of TorontoToronto, ON, Canada

**Keywords:** diacylglycerol kinase-ε, phosphatidylinositol cycle, lipid acyl chains, atypical hemolytic-uremic syndrome, re-entrant helix, arachidonic acid, rodents- and primates-specific signatures

## Abstract

In mammals there are at least 10 isoforms of diacylglycerol kinases (DGK). All catalyze the phosphorylation of diacylglycerol (DAG) to phosphatidic acid (PA). Among DGK isoforms, DGKε has several unique features. It is the only DGK isoform with specificity for a particular species of DAG, i.e., 1-stearoyl-2-arachidonoyl glycerol. The smallest of all known DGK isoforms, DGKε, is also the only DGK devoid of a regulatory domain. DGKε is the only DGK isoform that has a hydrophobic segment that is predicted to form a transmembrane helix. As the only membrane-bound, constitutively active DGK isoform with exquisite specificity for particular molecular species of DAG, the functional overlap between DGKε and other DGKs is predicted to be minimal. DGKε exhibits specificity for DAG containing the same acyl chains as those found in the lipid intermediates of the phosphatidylinositol-cycle. It has also been shown that DGKε affects the acyl chain composition of phosphatidylinositol in whole cells. It is thus likely that DGKε is responsible for catalyzing one step in the phosphatidylinositol-cycle. Steps of this cycle take place in both the plasma membrane and the endoplasmic reticulum membrane. DGKε is likely present in both of these membranes. DGKε is the only DGK isoform that is associated with a human disease. Indeed, recessive loss-of-function mutations in DGKε cause atypical hemolytic-uremic syndrome (aHUS). This condition is characterized by thrombosis in the small vessels of the kidney. It causes acute renal insufficiency in infancy and most patients develop end-stage renal failure before adulthood. Disease pathophysiology is poorly understood and there is no therapy. There are also data suggesting that DGKε may play a role in epilepsy and Huntington disease. Thus, DGKε has many unique molecular and biochemical properties when compared to all other DGK isoforms. DGKε homologs also contain a number of conserved sequence features that are distinctive characteristics of either the rodents or specific groups of primate homologs. How cells, tissues and organisms harness DGKε's catalytic prowess remains unclear. The discovery of DGKε's role in causing aHUS will hopefully boost efforts to unravel the mechanisms by which DGKε dysfunction causes disease.

## Introduction

**Figure 1:**
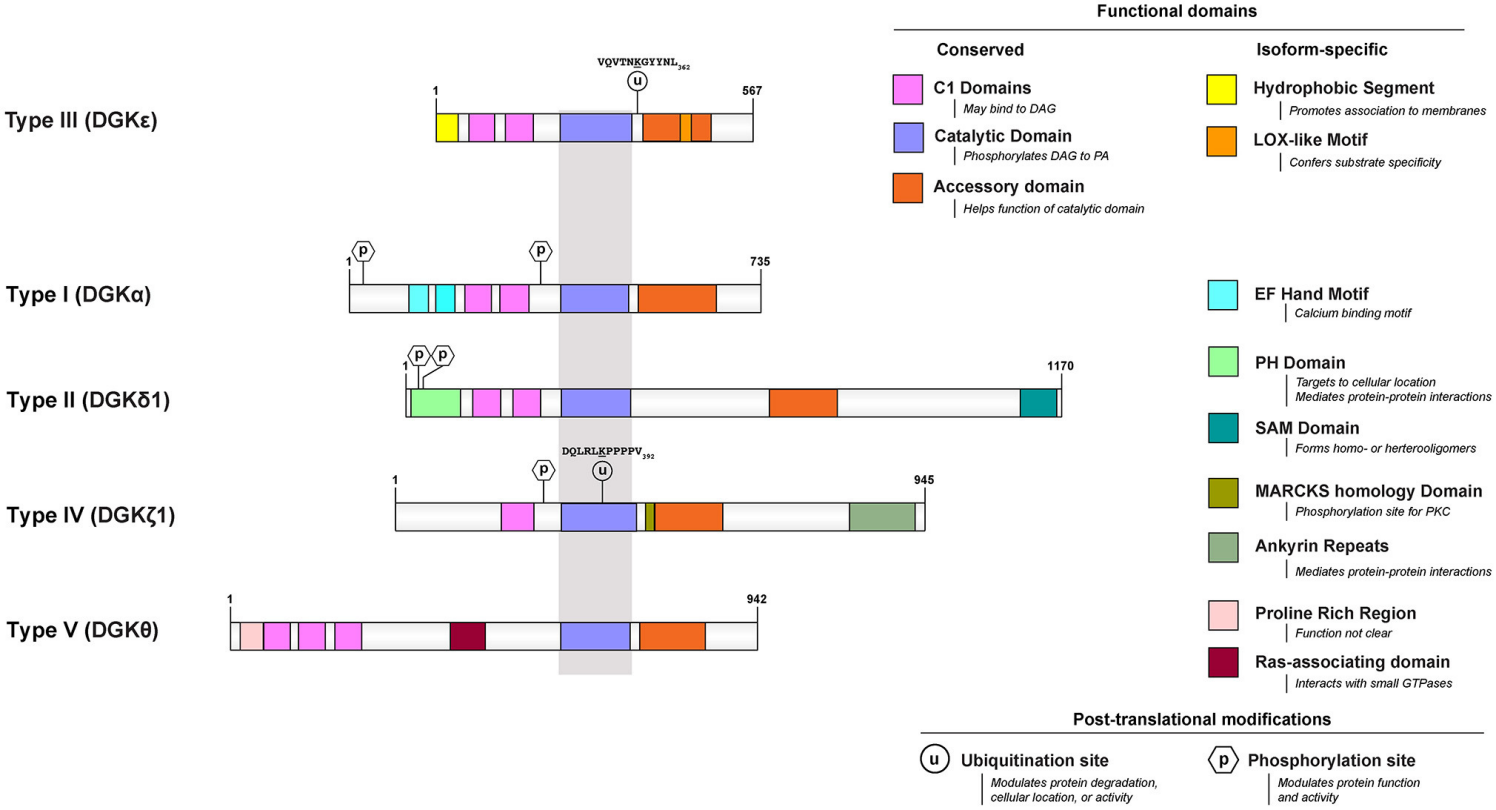
**Functional domains of DGKε are contrasted to that of prototypical DGKs from the other 4 subtypes**. All protein models were drawn to scale using the Illustrator for Biological Sequences (http://ibs.biocuckoo.org/) (Liu et al., 2015). The proteins are centered around the shared catalytic site (grayed area) because it is the most conserved feature across subtypes. Features that are unique to DGKε and other DGKs are listed on the right side. Known and/or possible phosphorylation (Shirai et al., 2012; Mertins et al., 2013; Park et al., 2015) and ubiquitination (Wagner et al., 2011; Komander and Rape, 2012; Mertins et al., 2013) sites are also displayed (see text and Table 1 for details). If there are more than one documented isoform, the characteristics of isoform 1 were used for each protein. The accession (and GI) numbers for the proteins illustrated are: DGKε, NP_003638.1 (4503313); DGKα, NP_958853.1 (41872494); DGK δ1, NP_003639.2 (25777596); DGKθ, NP_001338.2 (40806175); DGKζ1, NP_963290.1 (41872522).

Among the many isoforms and gene-splicing variants of mammalian DGK, the DGKε isoform is one of the most unique in its properties. DGKε is the smallest known isoform, it is the only one with no domain for binding a specific ligand, it is the only form that has a predicted transmembrane segment, and it is unique in having specificity for the acyl chain composition of the substrate. Mammalian DGKs can be divided into 5 types. DGKε is the only Type 3 isoform (Figure 1).

DGKε may also have a unique functional role in catalyzing one of the steps in the phosphatidylinositol-cycle (PI-cycle). The importance of the biological role of DGKε is suggested by the fact that it is the only DGK isoform that is associated with a human disease, namely atypical hemolytic uremic syndrome (aHUS).

## Interaction with diacylglycerol

### Atypical C1 domains

DGK catalyzes the reaction between DAG and ATP to produce PA and ADP. The C1 domains of protein kinase C bind DAG and phorbol esters directly via interactions mediated by select residues (Colón-González and Kazanietz, 2006). Amino acid sequence analysis revealed that all mammalian DGKs harbor at least two segments homologous to the prototypical C1 domain (Hurley et al., 1997). Multiple teams independently demonstrated that for most DGKs, these domains do not bind DAG or phorbol esters (Ahmed et al., 1991; Sakane et al., 1996); [DGKγ (Shindo et al., 2001, 2003) and DGKβ (Shindo et al., 2003) are notable exceptions]. Interestingly, some truncated DGKs devoid of all C1 domains have preserved catalytic activity (Sakane et al., 1996). Data suggest that DGKε may be unique among these DGKs: truncation resulted in complete abrogation of 1-stearoyl-2-arachidonoyl glycerol (SAG) phosphorylation (Tang et al., 1996). The biological function of these “atypical C1 domains” (Hurley et al., 1997) remains elusive to this day.

### Specificity for DAG with certain acyl chains

**Figure 2:**
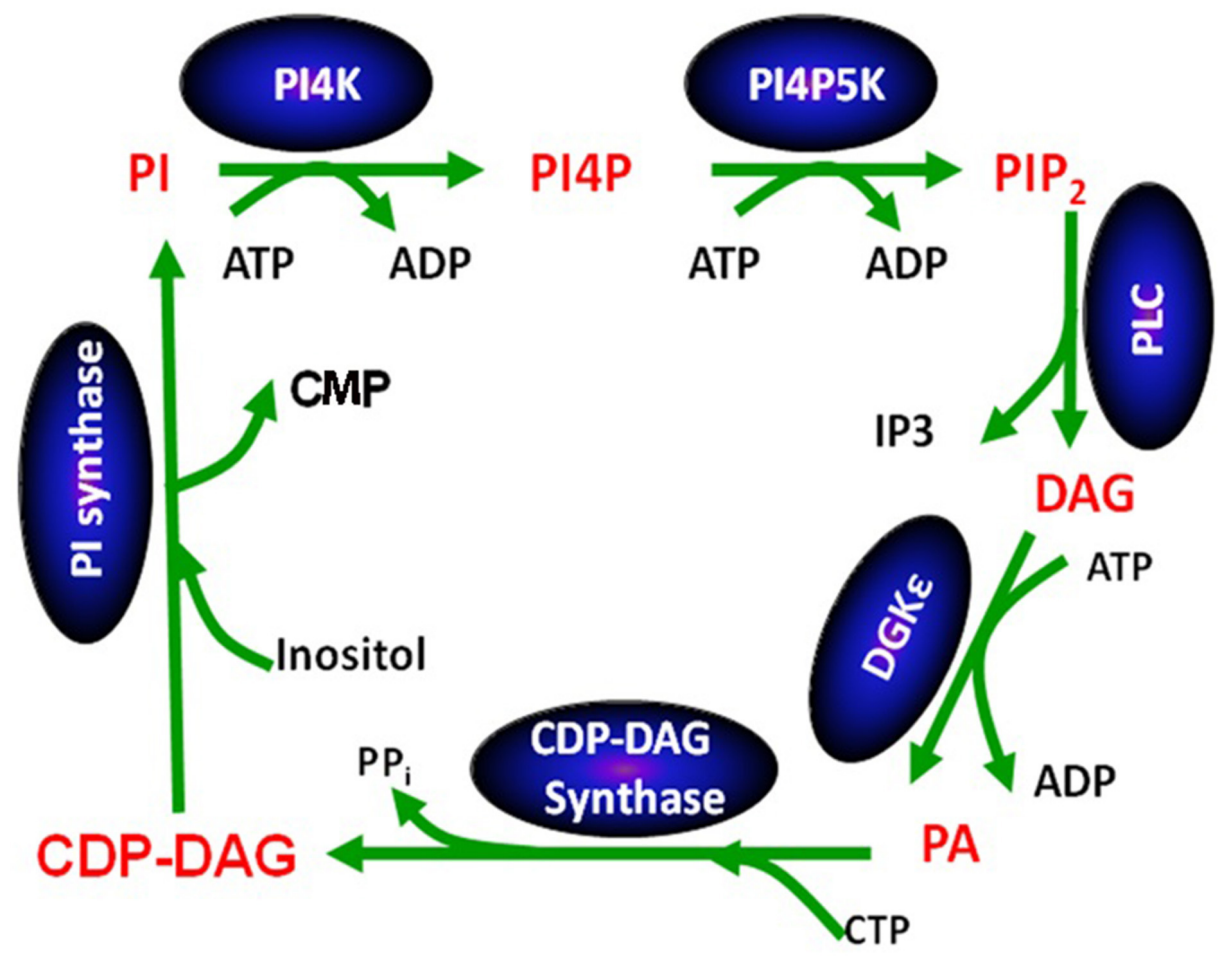
**The phosphatidylinositol cycle**. Enzymes involved in the catalysis of each step in the cycle are written in a blue oval above the arrow for the reaction using abbreviations. Each lipid intermediate in the cycle is written in red. Figure reproduced from Epand (2016).

Except for DGKε, all other isoforms of DGK phosphorylate DAG at rates that are largely independent of the nature of the acyl chains of DAG (Topham and Prescott, 1999). In contrast, the reaction catalyzed by DGKε is very sensitive to the acyl chains displayed by DAG: its peak activity is when the DAG substrate is SAG (Tang et al., 1996; Pettitt and Wakelam, 1999). The DAG molecule harboring these specific acyl chains is a critical lipid intermediate of the PI-cycle (Figure 2). In addition to DGKε, another enzyme that has specificity for lipid substrates with 1-stearoyl-2-arachidonoyl is CDP-diacylglycerol synthase 2 (CDS2) (D'Souza et al., 2014). The possible role of these two enzymes in enriching the lipid intermediates of the PI-cycle is discussed below. In addition, acyl chain remodeling of phosphatidylinositol (PI) through the Land's cycle, also contributes to acyl chain enrichment (Gijón et al., 2008).

#### *In vitro* detergent-based assays

Data supporting DGKε's exquisite substrate specificity remain incomplete. *In vitro* assays used to test its activity all rely on simultaneous co-solubilization with detergent of DGKε from cells together with DAG (Epand and Topham, 2007). A recent report suggests that the detergent used in these assays can exert a strong influence on the degree of substrate specificity (Natalini et al., 2013). The use of membrane bilayers in the form of liposomes would avoid possible artifacts caused by the presence of detergent and would more closely simulate a biological membrane. Until now, we have not been able to develop a liposome-based assay using extracts from cells overexpressing DGKε. However, we have recently succeeded in purifying human DGKε, thereby facilitating its incorporation into liposomes: this will be the first detergent-free enzyme activity assay for DGKε. We anticipate that the liposome-based assays will confirm the specificity of DGKε for SAG since this substrate specificity has also been demonstrated *in vivo* (see below) (Rodriguez de Turco et al., 2001; Milne et al., 2008).

#### *sn-2* arachidonoyl specificity

There have been more recent studies showing that the preference for an arachidonoyl chain in the *sn-2* position of DAG is very specific (Shulga et al., 2011a). The ability of several species of DAG having a stearoyl chain at the *sn-1* position and a polyunsaturated acyl chain at the *sn-2* position to act as a substrate for DGKε was determined. The activity of DGKε when presented with 18:0/20:4-DAG was ~5-fold higher than 18:0/18:2-DAG, and DGKε is unable to phosphorylate 18:0/22:6-DAG (Shulga et al., 2011a). On the basis of these results and others, it is clear that maximal activity requires that the DAG substrate have an arachidonoyl chain (20:4) at the *sn-2* position. We propose that the arachidonoyl group at the *sn-2* position fits into a specific binding site in DGKε.

#### *sn-1* stearoyl specificity

While an arachidonoyl group at the *sn-2* position is critical to DGKε activity by itself, it is not sufficient to make a good lipid substrate for DGKε. The monoglyceride 2-arachidonoyl-glycerol is a poor substrate for DGKε, and can even act as an inhibitor at higher concentrations (Gantayet et al., 2011). DGKε has essentially no activity in phosphorylating 1-monoacylglycerol substrates, but it does have 8% of the activity of SAG in phosphorylating 2-monoacylglycerol. However, this activity against monoglycerides, is not very different for DGKε compared with several other mammalian DGK isoforms (Sato et al., 2016). In addition, changing only the nature of the bond at the *sn-1* position of 1-SAG from an ester to an ether linkage (1-*O*-hexadecyl-2-arachidonoyl-*sn*-glycerol) also yielded a poor DGKε substrate (Epand et al., 2004).

The length of the fatty acyl chain at *sn-1* position also influences substrate quality: elongation from 18:0 to 20:0 led to reduced DGKε activity (70% from baseline). On the other hand, DGKε tolerates slightly shorter acyl chains at the *sn-1* position, as DGKε activity was 90% when substituting 18:0 for 16:0 (Lung et al., 2009). Introduction of unsaturation to the acyl chain at the *sn-1* position surprisingly also had only a small effect on the rate of substrate phosphorylation by DGKε. Thus, 20:4/20:4-DAG has about 80% the activity of SAG (Shulga et al., 2011a). Even more striking are the following observations: when compared to SAG, 18:0/18:2-DAG and 18:2/18:2-DAG generate respectively~20% and ~60% of the DGKε activity against SAG. Indeed, 18:0 is the optimal saturated acyl chain for the *sn-1* position when the *sn-2* position is occupied by 20:4, but not when the *sn-2* position is 18:2 (Shulga et al., 2011a). Thus, changing the linkage, length, or degree of saturation of the acyl chain at the *sn-1* position of DAG has substantial effects on substrate kinetics but many different acyl chains at the *sn-1* position result in substrates with substantial activity. We propose that the acyl chain at the *sn-1* position has an important, albeit less specific, interaction with DGKε that may indirectly affect the activity of the enzyme. For example, the fatty acyl chain at *sn-1* may modulate a physical property of the membrane surrounding the enzyme or influence the depth of insertion of DAG into the membrane. As a result, there appears to be a much less stringent requirement for the acyl chain at the *sn-1*, compared with the *sn-2* position of DAG.

### Catalytic accessory domain: homology to lipoxygenase sequence

We have shown that the most hydrophobic segment of DGKε, which is predicted to bind lipids, can be deleted without loss of enzymatic activity or specificity (Dicu et al., 2007; Lung et al., 2009). When searching for an alternative lipid binding site, we decided to explore a segment of DGKε's accessory domain that is homologous to the arachidonic acid binding site of lipoxygenase (Neau et al., 2009). The finding of such a homologous substrate binding site between lipoxygenase and DGKε was unexpected because the reactions catalyzed by these two enzymes are different, so is the chemical nature of the arachidonoyl groups of the substrates. Remarkably, no other mammalian DGK isoform harbors this novel motif, which is referred to as the “lipoxygenase (LOX)-like motif” (Shulga et al., 2011b).

This motif is characterized by a string of residues: L-X_(3−4)_-R-X_(2)_-L-X_(4)_-G, in which X_(*n*)_ is n residues of any amino acid. The critical residues are invariant through vertebrate evolution for both DGKε and lipoxygenase (Shulga et al., 2011b). They were identified on the basis that site-specific mutation results in the loss of enzymatic activity or arachidonoyl specificity. The most important reduction of DGKε activity was observed when leucine residues were substituted with more polar and/or sterically smaller amino acids; the impact of substitutions with less polar and/or sterically larger residues was not as dramatic. Most interestingly, substitution of a single amino acid converted DGKα to a LOX-motif-containing DGK that exhibited more specificity for arachidonoyl-containing DAG than unmodified DGKα (Shulga et al., 2011b).

Most mutations of key residues within DGKε's LOX motif resulted in a marked loss in catalytic activity. As a result, accurate assessment of the role of the LOX motif in directing the specific activity of DGKε against arachidonoyl-containing DAG species was challenging. We therefore proceeded to test the impact of mutating residues adjacent to the LOX motif on DGKε substrate specificity (D'Souza and Epand, 2012). This region, adjacent to the LOX motif is a hydrophobic segment contained within the “accessory domain” of DGKε. Unexpectedly, mutagenesis of several residues in this region of DGKε, which is also highly conserved in evolution, had a higher activity toward SAG when compared to wild-type. SAG was the best substrate for all mutants tested, followed by 1,2-diarachidonoyl glycerol. However, each mutant exhibited differences in the relative activity for different DAG substrates. For example, for the wild type enzyme the ratio of activity against 1-stearoyl-2-linoleoyl glycerol vs. SAG is 0.11. However, this ratio ranges from 0.03 to 0.22 for the 5 mutants tested. We conclude that these mutations perturb the lipid binding site, resulting in either enhanced or reduced substrate specificity (D'Souza and Epand, 2012).

## DGKε purification and stability

### Structural information is lacking for DGKε

There is currently no crystal structure available for any mammalian DGK isoform. Co-crystallization of DGKε with its substrate would be particularly informative. Altogether, these data would be invaluable to drive the discovery of isozyme-specific inhibitors, of which there is only one, for a different DGK isozyme (Liu et al., 2016). However, to do so requires a robust method for expressing and purifying large quantities of DGKε. In our hands, determining the optimal expression system has proven challenging. While bacteria express high levels of recombinant human DGKε, the enzyme recovered is not useful since it is inactive. Human DGKα was successfully expressed in yeast (Abe et al., 2003), but our attempts to express DGKε in the yeast *Pichia* were not successful.

We recently showed that insect (Sf21) cells are excellent bioreactors to produce high amounts of active recombinant DGKε (Prodeus et al., 2013). We recently succeeded to purify full-length and truncated (Δ40) human DGKε to near homogeneity using this cell system coupled to Nickel-affinity chromatography (Jennings, 2016). *In vitro* testing confirmed that both forms retained DGKε's substrate acyl chain specificity.

As mentioned in the Section Integral vs. Peripheral Membrane Protein, DGKε contains a putative membrane-spanning alpha helix at its N-terminus (Decaffmeyer et al., 2008). DGKε proteins generated using our protocol were instrumental in allowing us to test various hypotheses about the role of DGKε's amino terminus in binding to membrane lipids and its relevance to the overall activity and stability of the enzyme (Jennings, 2016).

### Glycerol stabilizes purified DGKε and DGKεΔ40 structure

Circular dichroism analysis of purified DGKε and DGKεΔ40 in solution shows that truncating the N-terminal α-helix does not impact the secondary structure of DGKε. Both forms of recombinant DGKε were noted to be highly unstable, losing enzymatic activity and secondary structure in a period of hours after purification. Experiments aimed at monitoring temperature-dependent loss of secondary structure indicates that both constructs undergo a biphasic transition from folded to unfolded states, with transitions occurring at ~56°C and ~77°C in a buffer containing 20% glycerol. We demonstrated that adding a high percentage of glycerol to the recombinant DGKε solutions had dramatic stabilizing effects (Jennings, 2016). We also showed that glycerol concentrations higher than 20% were necessary to facilitate the partial refolding of DGKε and DGKεΔ40 after thermal denaturation.

### Purified DGKε and DGKεΔ40 are active

Activity measurements of purified DGKε and DGKεΔ40 reveal that both constructs retain their acyl chain specificity for SAG (Jennings, 2016). These studies of activity reveal dramatic losses in activity following purification at room temperature, 4°C storage, −80°C storage, and particularly during cycles of freezing/thawing. The incorporation of glycerol into the purification of DGKε as well as during storage dramatically reduces but does not eliminate the observed losses in activity (Jennings, 2016). The absence of the N-terminal hydrophobic segment does not compromise specific activity in a detergent-phospholipid mixed micelle system and suggests that there are additional regions of DGKε that play critical roles in associating the protein to membranes/micelles. The advancements made in the purification and stabilization of DGKε and DGKεΔ40 will facilitate novel studies utilizing more biologically relevant liposome systems.

In contrast to measuring activity in detergent-phospholipid mixed micelle systems, liposome systems provide insight into how bilayer properties and specific lipid species affect enzyme function. A Ca^2+^-independent, water soluble DGK has been studied using liposomes (Thomas and Glomset, 1999a,b). However, DGKα and DGKζ are the only specific mammalian isoforms to be studied in a liposome-based system (Fanani et al., 2004). These enzymes were not purified; instead, they were recovered by salt extraction of cell pellets from mammalian cells overexpressing the particular DGK isoform (Fanani et al., 2004). Regardless, the cruder preparation still provided valuable information regarding the critical role of lipids in altering the activity and specificity of DGKα (Fanani et al., 2004). The successful purification of DGKε is facilitating similar studies in liposomes and will lead to novel findings regarding the activation/inhibition of this enzyme. Furthermore, the purification of DGKε is leading the way to more detailed studies of structure and protein interactions. It is also aiding the screening process for the discovery of a DGKε-specific inhibitor.

## DGKε has a role in the PI-cycle

The PI-cycle (Figure 2) has important roles in signal transduction and in lipid synthesis. Metabolic cycles have particular properties that are intrinsic to their cyclical nature. The concentrations of the PI-cycle intermediates quickly reach a steady-state, which then lasts over prolonged periods of time. The intermediates of the cycle are synthesized at the same rate that they are utilized and they are continually regenerated because they are intermediates within a cycle. The only members of the cycle that are likely to change with time are those that also are either substrates or products of reactions outside of the cycle. In general, the intermediates of the cycle, in addition to being substrates and products of reactions in the cycle, also function as catalysts for the cycle, since the functioning of the cycle neither creates nor destroys these intermediates. They increase the rate of interconversion among intermediates of the cycle, i.e., the rate at which the cycle “turns.” The PI-cycle has features that make it different from other metabolic cycles. First, it requires steps that are in two different membrane compartments, namely the plasma and endoplasmic reticulum (ER) membranes (Epand, 2016). DGKε is found in both the plasma membrane (Decaffmeyer et al., 2008) as well as in the ER (Kobayashi et al., 2007), targeted by the amino-terminal segment of DGKε (Matsui et al., 2014). Second, the lipid intermediates of the cycle are normally highly enriched with very specific lipid species that harbor 1-stearoyl-2-arachidonoyl fatty acyl chains.

It is interesting to note that DAG produced by phospholipase C-catalyzed hydrolysis of phosphatidylinositol-(4,5)-bisphosphate (PIP2) is highly enriched with SAG (Pettitt and Wakelam, 1999). This is peculiar because cell membranes contain many other types of DAG. SAG phosphorylation to PA catalyzed by DGKε is one of the key steps of the PI-cycle. It is followed by a series of conversions that ultimately lead to the synthesis of PI in the ER. This pathway includes another 1-stearoyl-2-arachidonoyl-specific enzyme, CDS2 (D'Souza and Epand, 2014). Since the other enzymatic reactions of the PI-cycle have no effect on the fatty acid chains, all intermediates share the 1-stearoyl-2-arachidonoyl backbone.

PI from DGKε-null cells contain less arachidonic acid (Milne et al., 2008) and stearic acid (Lung et al., 2009) than control cells. These data suggest a critical role for DGKε in determining the acyl chain composition of cellular phosphoinositides. Of interest, PI is much more affected than PA when taking into consideration changes in the acyl chain composition (Milne et al., 2008). This is a remarkable finding since PA is the direct product of the reaction catalyzed by DGKε. In contrast to normal cells, many cancer cells do not exhibit enrichment with 1-stearoyl-2-arachidonoyl-containing PIs, but rather are enriched with somewhat shorter and less unsaturated acyl chains (Naguib et al., 2015; Kimura et al., 2016). Differences between the PI-cycles of normal and cancer cells have yet to be thoroughly investigated: we anticipate that the activity of other DGKs and/or CDS enzymes must supplant that of DGKε/CDS2 during oncogeny (Epand, 2016). The fact that patients with complete DGKε deficiency do not appear to have increase cancer risks suggests that this mechanism is unlikely to be a primary driver (See Section Relationship to Disease).

## DGKε deficiency increases incorporation of glycerol into lipid

Recent studies suggest that DgkE^−/−^ mouse embryonic fibroblasts (MEFs) incubated with ^3^H-glycerol exhibit increased glycerol incorporation into various glycerolipids (Shulga et al., 2013). Preliminary studies from our laboratory suggest that this finding is likely due to increased glycerol kinase (GK) expression in Dgkε^−/−^ MEFs (So et al., 2016). We also found that these cells also consistently exhibit higher than normal p53 levels (So et al., 2016). We investigated this tumor suppressor in more detail because it is known to regulate GK expression (Goldstein et al., 2013). We thus propose that normally, DGKε is a negative regulator of glycerol incorporation through GK, via modulation of p53. In addition, p53 has been shown to exhibit various interactions with lipids. Links to p53 are likely to be complex since it also exhibits strong electrostatic interactions with lipids such as cardiolipin, phosphatidylglycerol, and PA *in vitro* (Li et al., 2010; Goldstein et al., 2013). In addition, its translocation from the nucleus to the mitochondria is modulated by CDS2 (the PI-cycle enzyme mentioned earlier) (Li et al., 2010). Finally, mutations in p53 have been linked to alterations in the acyl chain composition of PI species in a number of cell cultures of human and mouse origin (Naguib et al., 2015). Since p53 has many well-studied anti-tumorigenic roles in cells, DGKε-specific inhibitors may be useful as potential anti-cancer treatments. Such therapy would be expected to be most efficacious to treat p53-dependent brain cancers since DGKε is highly expressed in brain tissue (Shulga et al., 2011c). However, further studies are required to fully understand how DGKε interacts with p53, and how such an interaction might contribute to or prevent the progression of cancer.

## Integral vs. peripheral membrane protein

Membrane proteins are classified as peripheral or integral, based on their ease of extraction from a membrane. This empirical definition, which is based on experimental observations, is somewhat arbitrary since there is no fundamental difference between these two classes of membrane proteins. In reality, data suggest a continuum of small changes between peripheral and integral membrane-associated proteins. In many respects DGKε is an example of a protein that displays properties that are intermediary between these two protein types. For example, solubilization of overexpressed DGKε did not occur at neutral pH but partial solubilization occurred at alkaline pH (Dicu et al., 2007). It is well-known that varying the pH from neutral to alkaline should have no effect on the extraction of integral proteins from membranes (the structure of the membrane is unaffected by this change). Depending on the context, DGKε proteins may thus exhibit properties that are expected of integral or peripheral membrane proteins.

A number of predictive algorithms suggest that DGKε residues 20–40 can form a transmembrane helix (Glukhov et al., 2007; Jennings et al., 2015). DGKε is the only mammalian DGK isoform with such a putative transmembrane segment. DGKε is also predicted to be the only DGK isoform that is permanently associated with the membrane (Glukhov et al., 2007). Several experimental lines of evidence support this hypothesis. First, a model peptide derived from this DGKε segment, flanked with strings of positively charged lysine residues, was shown to interact with anionic membranes (Glukhov et al., 2007). Third, during synthesis of the protein, glycosylation sites at the amino terminus of DGKε are exposed to the lumen of the ER (Nørholm et al., 2011). These data strongly support the notion that DGKε's hydrophobic segment can form a *bona fide* transmembrane helix in cells.

**Figure 3:**
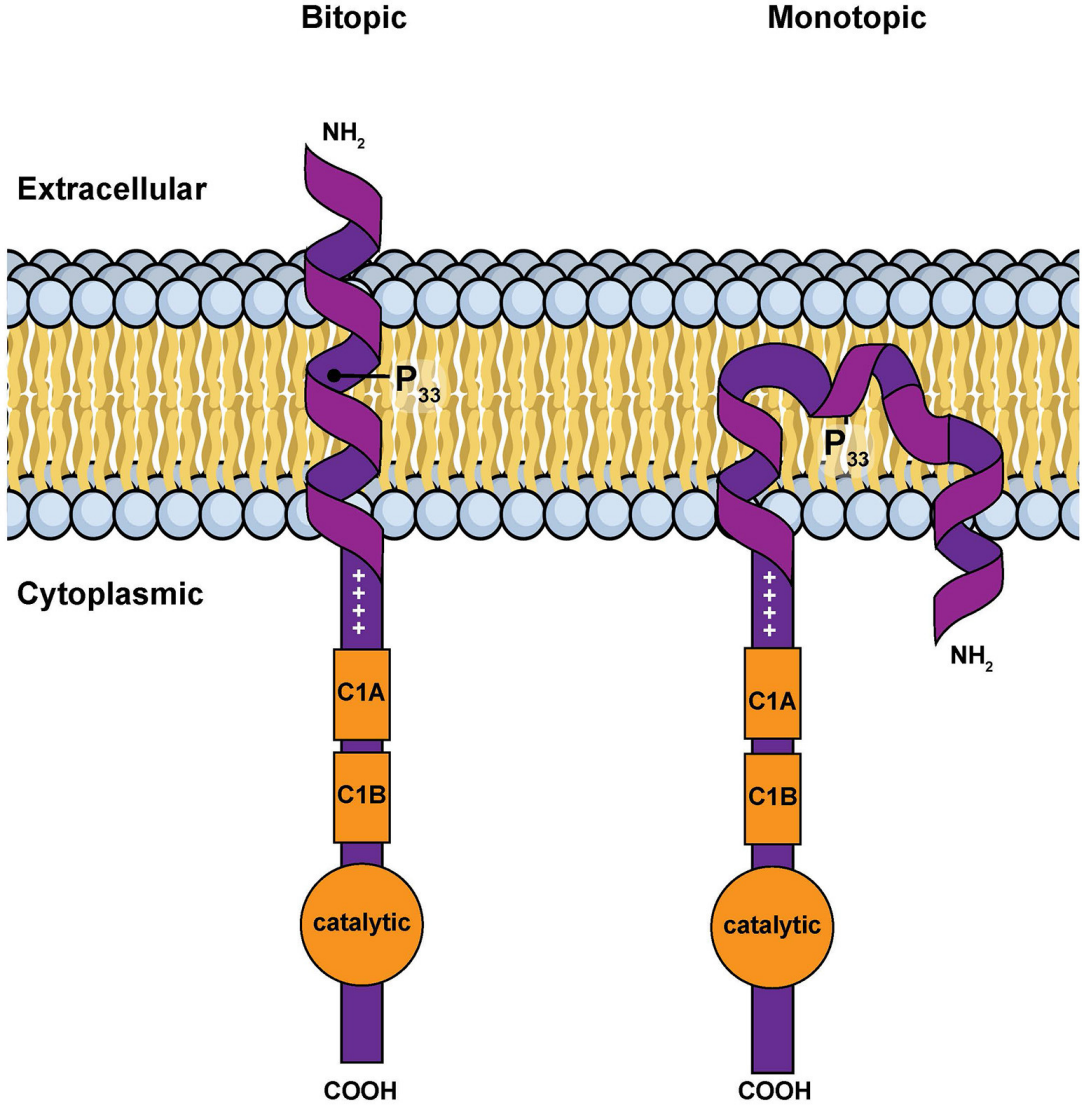
**Simple models for the amino terminal segment of DGKε inserted into a membrane either as a bitopic transmembrane helix or as a monotopic re-entrant helix (Nørholm et al., 2011)**. This illustration is a modified version of Figure 2 from an article on intracellular protein topogenesis (Blobel, 1980). The approximate position of Pro^33^, a residue important for the interconversion between these two conformations, is shown. The topological relationships of the intra and extracellular sides of the membrane bilayer, the location of the C1 domains and of the catalytic site are illustrated.

However, *in silico* calculations suggest that this hydrophobic segment has two possible stable conformations when associated with a membrane: a classic transmembrane helix or a U-shaped, re-entrant helix that enters and leaves the membrane on the same side of the bilayer (Decaffmeyer et al., 2008). If the hydrophobic segment is transmembrane, parts of the amino terminus of DGKε would be expected to be exposed to the extra-cellular environment (Figure 3). We tested for this possibility by using a DGKε construct with a FLAG-tag added to the amino terminus. We reasoned that immunofluorescence detection of FLAG-DGKε in non-permeabilized cells should only be possible if the amino terminus crosses the plasma membrane. We showed that permeabilization was required for visualization of FLAG-DGKε, strongly suggesting that DGKε forms mostly re-entrant helices (Decaffmeyer et al., 2008). Interestingly, FLAG-DGKε was detected in non-permeabilized cells after Pro33Ala mutagenesis; this proline residue is predicted to be key to form the U-shaped re-entrant helix [note that in earlier papers, such as reference (Decaffmeyer et al., 2008), we refer to this proline residue as 32, corresponding to its numbering after cleavage of the N-terminal methionine to form the mature protein. In the present article residue numbering includes the N-terminal methionine as residue 1] (Decaffmeyer et al., 2008). The result also suggests that some DGKε locates to the plasma membrane. The presence of DGKε at the plasma membrane was demonstrated by Western blotting of an affinity-purified plasma membrane fraction from 3T3 cells that had been transfected with FLAG-DGKε (Decaffmeyer et al., 2008). Note that this proline residue is invariant in evolution (See Supplementary Materials), suggesting that it plays an important functional role, perhaps by allowing facile interconversion between transmembrane and re-entrant helical conformations.

There is thus conflicting evidence as to whether the amino terminal segment of DGKε forms a transmembrane or a re-entrant helix in cell membranes. Mathematical modeling reveals that the energy difference between these two distinct conformations is small (Decaffmeyer et al., 2008). It is thus possible that one conformation may predominate depending on the discrete properties of the surrounding lipid bilayer (e.g., membrane thickness, charge, the intrinsic curvature of the monolayers, or the presence of specific lipids). Interestingly, many of these parameters differ significantly when the plasma and ER membranes are compared. Our results may not be contradictory, but rather reflect normal comformational changes dictated by environmental conditions or local topogenic signals.

## DGKε interactome

No systematic investigation has focused on identifying the array of proteins that interact with DGKε. The only data available in that regard are from two studies done in HEK293T cells. In the first study, the investigators use immunoprecipitation followed by mass spectrometry (LC-ESI-MS/MS) to delineate the interactomes of 338 Flag-tagged bait proteins, including DGKε. They reported that DGKε pulled down several proteins with no clear links to known DGK biology (see Table 1 for details): CDCA1, NUDC, NUF2, PAICS, PDHA1, SET (Ewing et al., 2007). The other study used a similar mass spectrometry-based approach to identify the interactomes of arrestinβ1 (ARRB1) and arrestinβ2 (ARRB2), which are important negative regulators of G protein-coupled receptors (GPCR) (Premont and Gainetdinov, 2007). It revealed that DGKε was associated with both ARRB1 and ARRB2, and that these interactions were not modulated by GPCR activation (Xiao et al., 2007). In yet another proteome-wide interactome study performed on liver cell extracts, DGKε was found to interact with MRPL44, a protein involved in mitochondrial ribosomes (Table 1).

**Table 1:** **Data on the possible DGKE interactome abstracted from three proteomics studies**.

**Bait (OMIM No.)**	**Prey (OMIM No.)**	**Full gene name**	**Other names**	**Known functions**
DGKε^a^ (601440)	PAICS (172439)	Phosphoribosylaminoimidazole carboxylase	AIRC	Purine biosynthesis
	NUDC (610325)	Nuclear distribution protein C homolog	–	Nuclear movement protein that associates with dynein
	CDCA1 (611772)	Cell division cycle-associated protein 1	NUF2	Chromosome segregation and the spindle checkpoint
	PDHA1 (300502)	Pyruvate dehydrogenase, alpha-1	PDHCE1A, PDHE1A, PDHA	Conversion of pyruvate into acetyl-CoA
	SET (600960)	Suppressor of variegation, enhancer of zeste, and Trithorax	IGAAD	Inhibitor of protein phosphatase 2A
DGKε^b^ (601440)	MRPL44 (611849)	Mitochondrial ribosomal protein l44	–	Mitochondrial ribosome
ARRB1^c^ (107940)	DGKε (601440)	Arrestin, beta, 1	ARRB1, BARR1	G protein-coupled receptor desensitization
ARRB2^c^ (107941)	DGKε (601440)	Arrestin, beta, 2	ARRB2, BARR2	G protein-coupled receptor desensitization

a *HEK 293 cells were transfected with flag-tagged bait (DGKε), followed by immunoprecipitation with anti-Flag antibody. The protein complexes associated with DGKε were analyzed by Mass spectrometry. DGKε was one of 338 baits (Ewing et al., 2007)*.

b *Liver cells (Wang et al., 2011)*.

c *HEK 293 cells were transfected with flag-tagged bait, followed by immunoprecipitation with anti-Flag anti-body. The protein complexes associated with the bait were analyzed by Mass spectrometry (Xiao et al., 2007)*.

These data should be considered as weak evidence of interactions since none of the studies offered independent experimental confirmation. It would have been reassuring to see at least one of the ARRBs in the list of DGKε interactors from the first study given that both studies used the same cells (HEK293T). No subsequent studies have sought to explore the physiologic relevance of these potential DGKε partners. Given DGKε's presumed regulatory role in the signaling pathway of PLC-activated by GPCR, the interactions with the ARRBs are by far the most promising. It is clear that important clues regarding the functions of DGKε in various mammalian cells could be gleaned from studies focused on the DGKε interactome. While the same mass spectrometry-based methodology could be used, other approaches such as BioID (Roux et al., 2013) should also be considered.

## Post-translational modifications of DGKε proteins

The extent to which post-translational modifications modulate DGKε function is yet another area of DGKε biology that is understudied. While phosphorylation of several DGKs has been demonstrated experimentally (Shirai et al., 2012), there is no evidence that it plays a major role in modulating DGKε function. Two studies that presented comprehensive phosphorylation data from human samples confirmed that DGKε phosphorylation levels, if present, must be low (Mertins et al., 2013; Park et al., 2015).

Several proteome-wide screens focused on other post-translational modifications have been published in recent years. The most striking finding is that two distinct studies uncovered the exact same ubiquitination site at lysine 357 in human DGKε (Wagner et al., 2011; Mertins et al., 2013). The cells used in the studies included HEK293T (human embryonic kidney) and MV44-11 (acute monocytic leukemia) in one study, and Jurkat cells (immortalized human T cells) in the other. The same modification was also found at the homologous murine locus, at lysine 354 (Wagner et al., 2012). In the mouse, Dgkε ubiquitination was only observed in proteins extracted from the brain (other tissues tested that did not express ubiquitinated Dgkε included heart, liver, kidney and muscles). This remarkable tissue specificity may be unique to mice since three non-brain cell types were used for the human studies. The so-called “ubiquitin code” is known to control a wide array of biological functions, including enhanced degradation, targeting to specific cellular location, modulation of function, and regulation of protein-protein interactions (Komander and Rape, 2012). It will be important to determine the relevance of ubiquitination at this site and to determine if tissue- and species-specific patterns exist.

## Novel sequence features of DGKε

**Figure 4:**
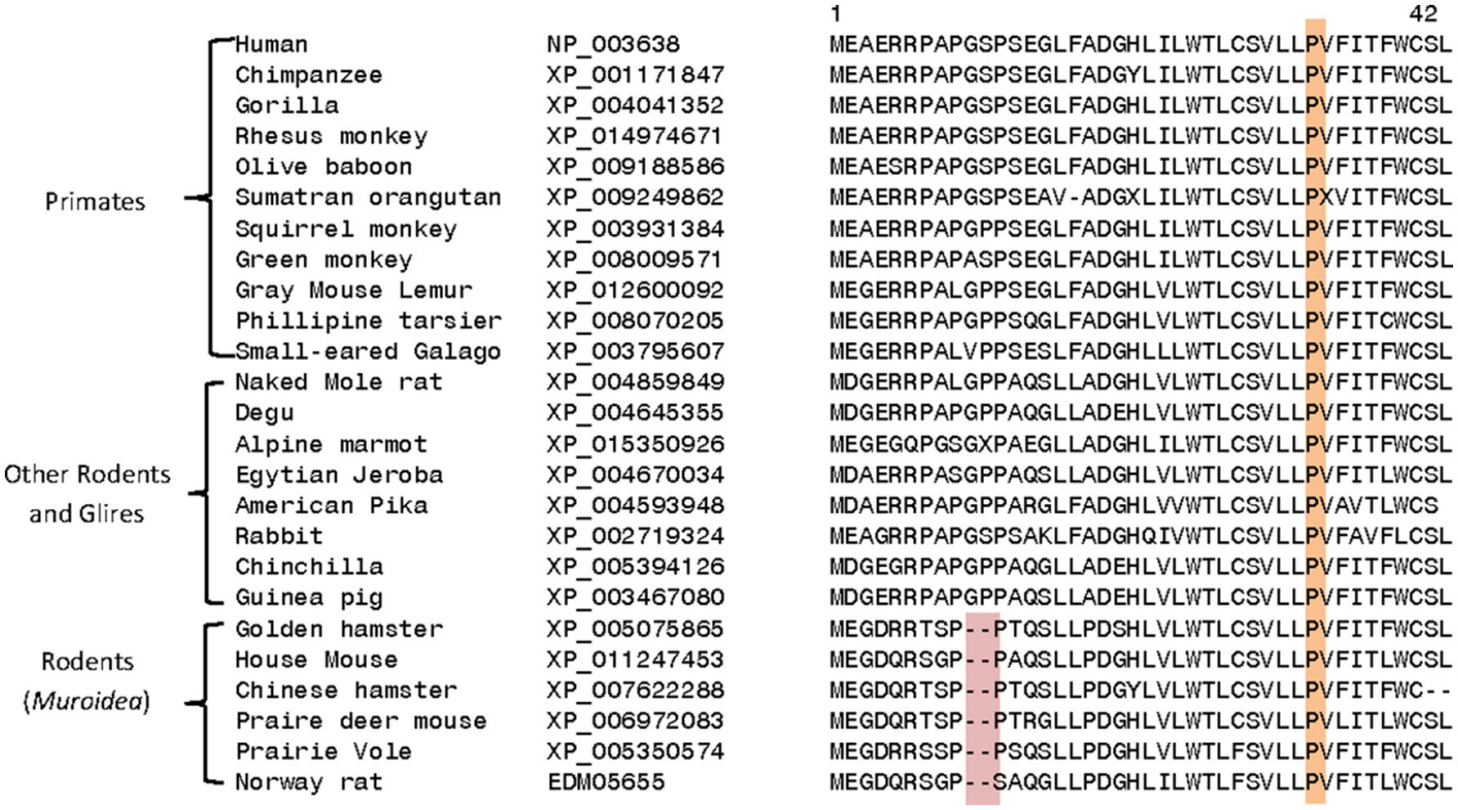
**Excerpts from a multiple sequence alignment of DGKε sequences showing two conserved characteristics that are of interest**. Protein sequences of DGKε homologs were obtainedfrom the NCBI database (http://www.ncbi.nlm.nih.gov/) and the conserved characteristics noted here were identified in their sequence alignment as previously described (Gupta, 2016). The first characteristic (highlighted in pink) consists of a 2 aa deletion in a conserved region of DGKε that is a unique property of the Muroidea family of rodents, which includes rats, mice and hamsters, but not found in the primates or other mammalian homologs. The sequence information is shown for all rodents and primate homologs and a limited number of other species. The sequence alignment also shows that the proline residue present at position 33 in human DGKε, which is implicated in the re-entrant property of the N-terminal helical region, is a conserved property of all vertebrate DGKε sequences (see also Supplementary Table 1).

Homologs exhibiting a high degree of sequence similarity to DGKε are present in most eukaryotic organisms, except plants, fungi, and some unicellular organisms. Comparison of DGKε sequences from mammalian species reveals a number of interesting differences that are specific for particular groups of animals. For example, the DGKε homologs from the *Muroidea* family of rodent species (e.g., rats, mice, hamsters, gerbils; Catzeflis et al., 1992) contain a conserved 2 amino acid deletion near the N-terminus, which is not found in the homologs from other mammals (Figure 4). Conserved inserts and deletions in protein sequences play important roles in mediating novel protein-protein or protein-ligand interactions (Akiva et al., 2008; Singh and Gupta, 2009). Thus, it is likely that this rodent-specific genetic alteration may also affect the biological function of the rodent DGKε in some subtle manner. The functional significance of this alteration may become clearer as the role of the amino terminus is better understood.

**Figure 5:**
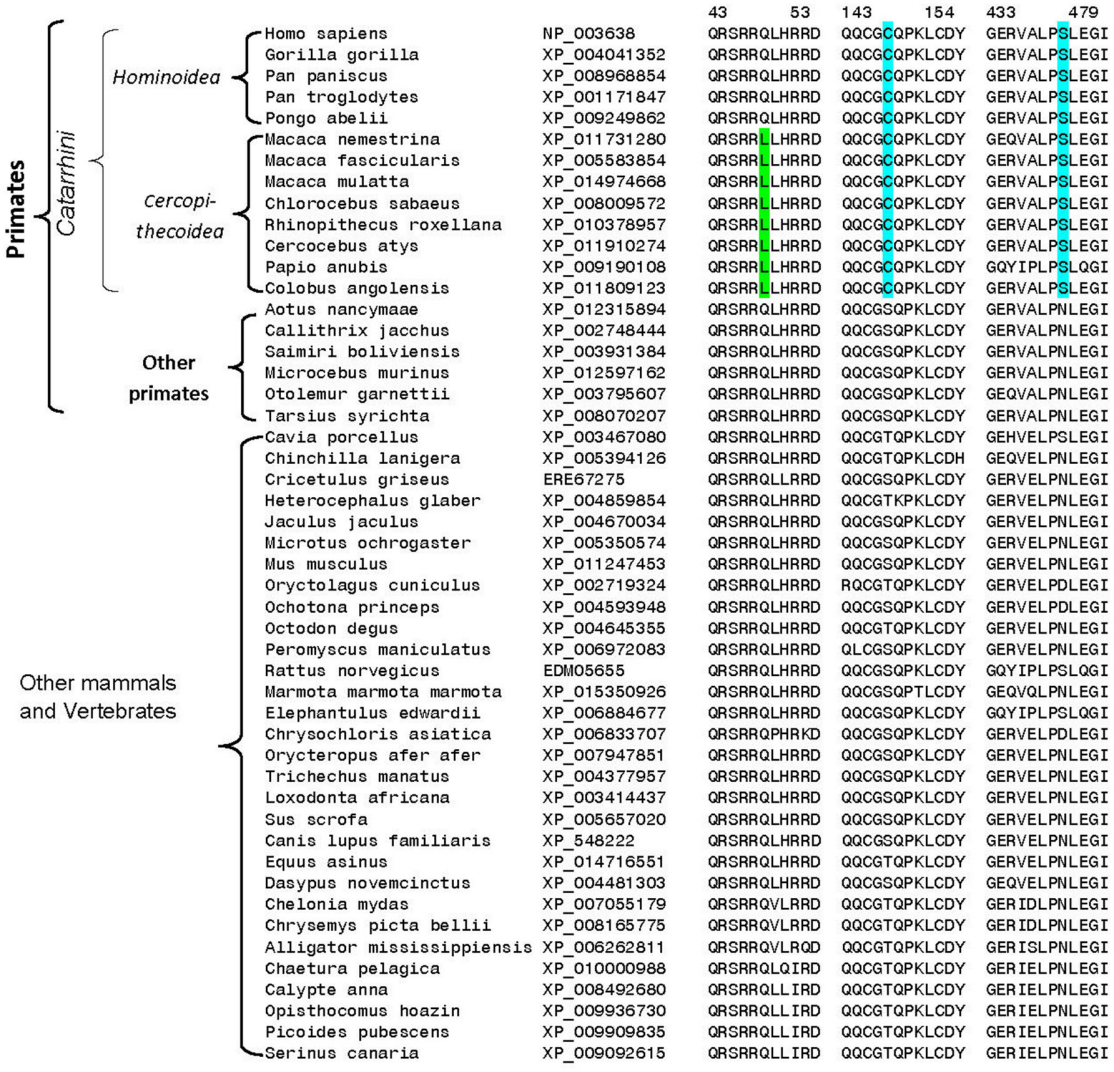
**Excerpts from multiple sequence alignment of DGKε sequences showing three conserved sequence polymorphisms that are specific for different groups of primates**. All of the sequences were downloaded from the NCBI database and the sequence alignment to identify conserved characteristic was carried out as in earlier work (Gupta, 2016). The two sequence polymorphisms (highlighted in blue) are specific for the Catarrhini subdivision of primates, which includes humans, great apes, gibbons, and old world monkeys. The third polymorphism marked in green is specific for the *Cercopithecoidea* family, comprising of the old-world monkeys. Sequence information is shown for all primate homologs and for representative species from other vertebrate groups.

A number of other specific changes seen in the DGKε homologs are specific for the Catarrhini subdivision of primates, which includes humans, great apes, gibbons, and old world monkeys (Figure 5). At positions 147 and 440 in the human DGKε, homologs from the Catarrhini subdivision contain cysteine and serine residues, respectively, whereas all other mammalian species have serine/threonine or aspartate/asparagine at these positions. Another specific change in the DGKε homologs present at position 48 is a distinctive characteristic of the old world monkeys (*Cercopithecoidea)*. All DGKε homologs from the *Cercopithecoidea* family contain a leucine in this position instead of the glutamine found in all other vertebrates. The high degree of specificity of the noted genetic changes within the indicated groups suggests that these changes are under strong selection pressure and may thus confer some as yet unknown biological advantages to DGKε functions in these primates.

**Figure 6:**
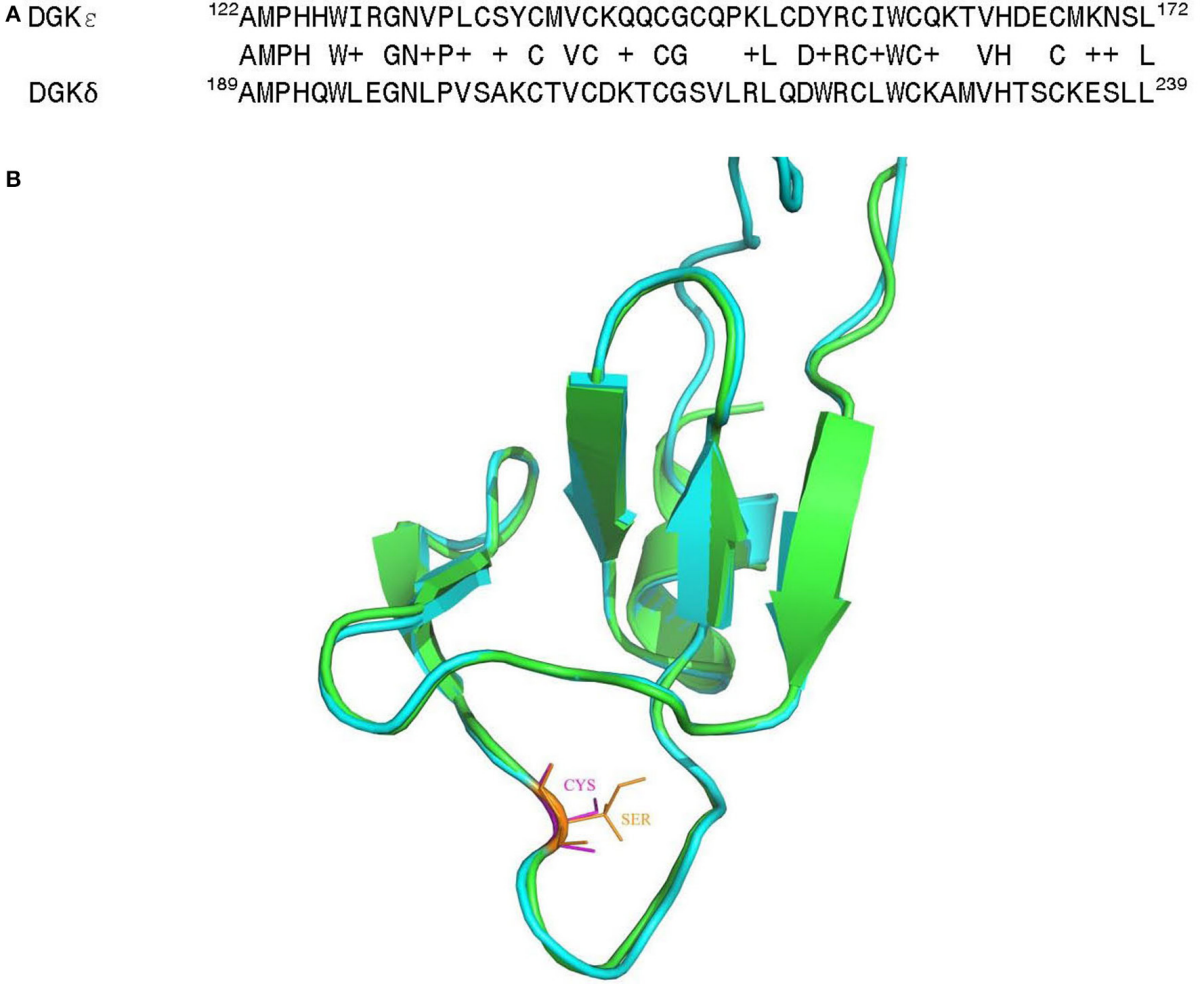
**(A)** Sequence alignment of the C1B domain from human DGKε with the corresponding region of the DGKδ isoform, whose solution structure has been determined by NMR (Miyamoto et al., 2004). **(B)** Superposition of the three-dimensional (3D) structures of the C1B domain of human DGKε (shown in green) with human DGKδ (shown in cyan). Homology models of the human DGKε were built using the NMR structural information for human DGKδ (PDB ID: 1R79) (Liu et al., 2015). The first model in NMR was utilized as the template and the structure was generated using MODELLER v 9.15 (Sali and Blundell, 1993). The structural comparison of the modeled human DGKε with the DGKδ isoform was carried out using PyMol (http://www.pymol.org).

It is of much interest that one of the above noted genetic changes at position 147 is located within one of the C1 domains (C1B) of DGKε. These are highly conserved and cysteine-rich domains that are typically involved in binding of DAG or phorbol esters (Hurley et al., 1997; van Blitterswijk and Houssa, 2000; Sakane et al., 2007; Jennings et al., 2015) (however, see also Section Atypical C1 Domains). As such, they may play a central role in directing the function of DGKs in cells. Alignment of the C1B domains for human DGKε and DGKδ reveals very high degree of sequence homology (Figure 6A). Based on the structural information for the most predominant form of DGKδ (Miyamoto et al., 2004), we have created and optimized a homology model of the C1B domain for human DGKε (Figure 6B) (Sali and Blundell, 1993; Shen and Sali, 2006; Xu and Zhang, 2011). The superposition of the modeled structure with the structure of DGKδ shows very high degree of structural homology between the two isoforms in the C1B domain and it also shows the location where the Cys substitution specific for the Catarrhini subdivision has occurred. It is possible that this additional cysteine primes the C1 domains to be more avid for DAG-like substrates. This hypothesis can only be confirmed via mutagenesis experiments and by solving the crystal structure of DGKε with its DAG substrate.

Another remarkable finding relates to the near-complete conservation of the hydrophobic segment (positions 21–42) in vertebrate species, including the proline residue at position 33. As discussed in the Section Integral vs. Peripheral Membrane Protein, this segment is hypothesized to act as a membrane-associated domain (Jennings et al., 2015). This proline residue is contained within the very hydrophobic segment near the amino-terminus of DGKε (see hydropathy plot of Figure 1 in Jennings et al., 2015). In this scenario, the proline residue at position 33 plays an important role in the formation of a re-entrant helix (Decaffmeyer et al., 2008). *In vitro* experiments have shown that mutagenesis of this position to alanine results in higher affinity for membranes via conversion of the re-entrant helix to a transmembrane helix (26). This suggests that there might be substantial functional advantage for DGKε to form a re-entrant helix so as to restrict its interactions with lipid bilayers to the inner leaflet. The sequence alignment of DGKε from various vertebrate species (mammals, amphibians, reptiles, birds, and fishes) and also some invertebrates shows that the proline at position 33 is completely conserved and invariant in all examined species (see Supplemental Figure 1). This in turn suggests that the re-entrant potential of this hydrophobic segment is an evolutionarily conserved property of the DGKε from different species.

## *In vivo* evidence from DGKε knock-out mouse

A Dgkε-null mouse was reported in 2001 by investigators that were studying the role of DGKs in the brain (Rodriguez de Turco et al., 2001). No major anomalies were noted despite extensive phenotyping that mostly focused on the neurological system (Rodriguez de Turco et al., 2001). Quantitative phospholipid studies on brain tissue from the Dgkε-null mice revealed deficits in arachidonic-acid containing PIP2, but no change in DAG or PA (Rodriguez de Turco et al., 2001). Similar studies in fibroblasts derived from these mice revealed a similar pattern (Milne et al., 2008). *In vitro* corroboration of these results with RNA interference has not been reported.

While exon 1 was unquestionably replaced by a neomycin resistance cassette in these mice (Rodriguez de Turco et al., 2001), it is important to acknowledge that absence of Dgkε protein has never been specifically demonstrated. The main reason for this omission is the poor performance of anti-murine Dgkε antibodies when used to detect endogenous Dgkε with Western blotting or tissue staining. It was also thought that even if made, this truncated version of DGKε, which would lack both C1 domains (tDGKε), would be unlikely to be functional (although, as mentioned in the Section Atypical C1 Domains, the role of these C1 domain remains unclear). Data obtained with another truncated DGKε (DGKεΔ, lacking the segment from Pro_6_ to Pro_188_) showed that such a truncated protein had no activity toward SAG (Tang, 1999). Of note, DGKεΔ activity toward other DAG substrates was not tested.

Now that work on Dgkε-null mice has potential health-related ramifications (for patients with DGKE-associated nephropathy, See Section Relationship to Disease), it is critical to determine if this model accurately reflects true Dgkε-deficiency. Indeed, incomplete knockouts have been described before, and the methodology employed to generate early mouse models often involved deleting exon 1 (Müller, 1999). According to the Knockout Mouse Project initiative (KOMP), the critical exon for DGKε is exon 3, not exon 1. KOMP is a major NIH-sponsored initiative aimed at making thousands of knockout mice widely available (Skarnes et al., 2011). Targeting of the critical exon maximizes the likelihood of generating a true knockout because this exon must be comprised in all spliced isoforms and, when deleted, it must create a frameshift mutation.

If expressed, tDgkε produced from a putative alternative start codon would be missing the C1 domains. Since the function of these atypical C1 domains is unclear (see Section Atypical C1 Domains), the functional impact of that loss is difficult to predict. Importantly, the kinase and catalytic accessory domains would be intact. tDgkε would thus resemble bacterial forms of dgks, which lack the segment harboring the C1 domains. Just like these dgks, tDgkε could in principle actively phosphorylate many other targets besides SAG, including other types of DAG (Walsh et al., 1990). More concerning however is the possibility that tDGKε may gain the ability to phosphorylate ceramide (Schneider and Kennedy, 1973). The cells may thus be simultaneously exposed to both a loss and a gain of function. The biological consequences of such a complex system would be challenging to interpret: it remains an open question whether this is a viable model for a human disease caused by a “simple” DGKε deficiency. For these reasons, it is critical to confirm if this mouse model is indeed a true knockout.

## Relationship to disease

### Atypical hemolytic uremic syndrome (aHUS)

**Figure 7:**
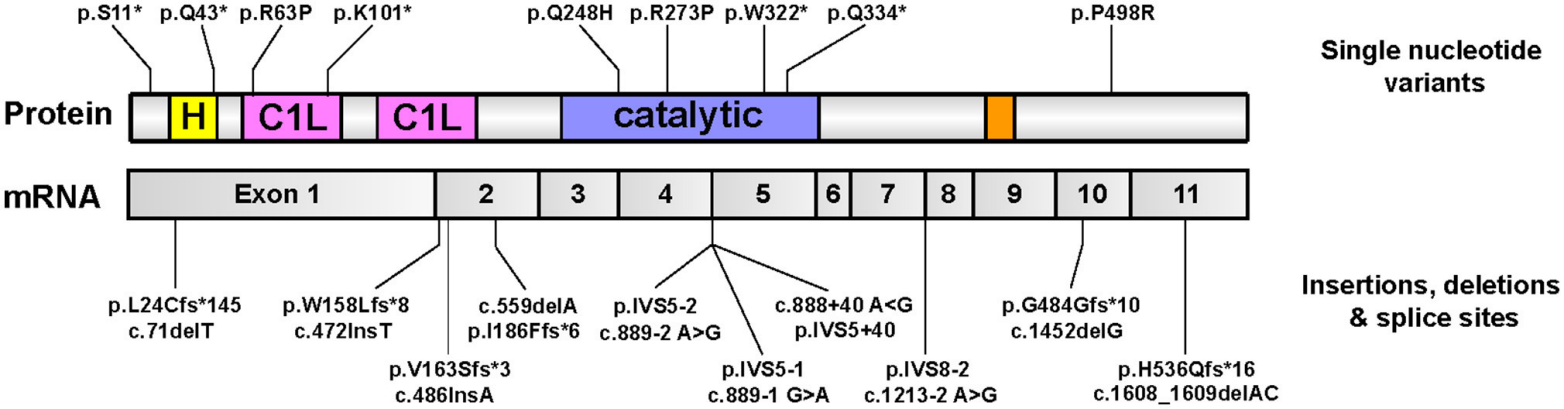
**Pathologic mutations in DGKE identified in patients with DGKE-associated nephropathy**. All published missense or nonsense Dgkε mutations that are reported in the literature (Lemaire et al., 2013; Ozaltin et al., 2013; Sánchez Chinchilla et al., 2014; Westland et al., 2014; Lee et al., 2015; Mele et al., 2015; Miyata et al., 2015), as of August, 2016 are overlayed on an illustration of Dgkε and its functional domains (top). Mutations that affect splice sites or result in insertions/deletions are overlayed onto an illustration of DGKε mRNA with the boundaries of the 11 coding exons (bottom). Mutation names used adhere to the standard method promulgated by the Human Genome Variation Society (HGVS) for sequence variation nomenclature (http://varnomen.hgvs.org). Single letter codes for amino acids are used. Abbreviations used: “c.”, cDNA position; fs, frameshift; IVS, intervening sequence (intron); “p.”, protein position.”

Whole-exome sequencing recently uncovered an unexpected link between homozygous mutations in the gene encoding for DGKε and DGKε-associated nephropathy (Lemaire et al., 2013; Ozaltin et al., 2013). The bulk of mutations are expected to result in complete loss-of-function (nonsense, splice site, frameshift); no clustering to any particular domain was observed (Figure 7). This rare condition is due to recurrent episodes of thrombosis in the kidney glomeruli microvasculature. The salient clinical findings are acute renal failure, low platelets, and hemolytic anemia. On that basis, it is hypothesized that DGKε protein must play an important role in regulating thrombosis in the human kidney. This discovery forced experts to reconsider the pathophysiologic underpinnings of aHUS (Quaggin, 2013). Of note, a small group of patients with pathogenic DGKE mutations present with clinical features that resemble more that of another glomerular disease, membranoproliferative glomerulonephritis (Ozaltin et al., 2013). Up until then, abnormal activation of the alternative complement pathway was thought to be invariably associated with nearly all forms of aHUS (Noris et al., 2012): the vast majority of aHUS patients harboring DGKE mutations exhibit no evidence of complement activation (Lemaire et al., 2013; Ozaltin et al., 2013). Only supportive measures may be offered to these patients because there are no targeted therapies.

DGKε was originally cloned from human umbilical vein endothelial cells, but mRNA expression was predominant in testes (Tang et al., 1996). The discovery of the link between DGKε and aHUS recently prompted more in-depth investigations focused on its presumed role in kidney biology. DGKε protein was shown to be expressed in three cell types that play major roles in kidney glomeruli, namely endothelial cells, podocytes, and platelets (Lemaire et al., 2013; Ozaltin et al., 2013). However, the lack of disease recurrence in patients after kidney transplantation strongly suggests that platelets, which are produced by the bone marrow, are unlikely to be central players in the disease process (Lemaire et al., 2013). The mechanism by which DGKε deficiency causes thrombosis exclusively in the kidney remains unclear. Its expression in other vascular beds has not been investigated. Quantification of key members of the PI-cycle needs to be assessed specifically in endothelial cells to determine if DGKε deficiency in this setting also leads to paucity of PIP_2_ (see Section DGKε Has a Role in the PI-Cycle). Experiments done in cultured endothelial cells show that siRNA knockdown of DGKε was associated with several phenotypes: endothelial cell increased activation, increase apoptosis and decreased proliferation (Bruneau et al., 2015). It will be important to determine if DGKε-null endothelial cells display the same characteristics because the siRNA knockdown and knockout of the same gene may yield very different phenotypes (Rossi et al., 2015).

The generation of an animal model is often very useful to delineate the biology of human diseases. Since most patients are expected to have DGKε deficiency, the Dgkε-null mouse reported in 2001 would be an ideal candidate model. The original report showed that there were no major abnormalities with these animals (see Section Post-translational Modifications of DGKε Proteins; Rodriguez de Turco et al., 2001). The renal phenotype of this mouse model was recently re-evaluated in more detail: the animals developed mild signs of renal disease with age (Zhu et al., 2016). Interestingly, glomerular lesions were noted in Dgkε-null mice after exposure to doses of nephrotoxic serum that did not affect wild type littermates (Zhu et al., 2016). Mouse models of aHUS often require exposure to exogenous triggers to reveal their pathogenic potential (Pickering et al., 2006; Thurman et al., 2012; Vernon et al., 2016). Importantly, exogenous factors are also known to act as triggers in many patients with genetic forms of aHUS (Kavanagh et al., 2013). Dgkε-null mice may thus be a promising research tool to further our understanding of DGKε-associated nephropathy (assuming it is a full knockout—see Section Post-translational Modifications of DGKε Proteins).

Data from the recent report focused on the Dgkε-null mice suggests that DGKε deficiency leads to systemic inability to induce cyclooxygenase-2 (Cox-2) (Zhu et al., 2016). This enzyme, which is responsible for inducible production of prostanglandins, was previously shown to be decreased in the brains of Dgkε-null mice after kindling (Lukiw et al., 2005). When exposed to lipopolysaccharides, wild-type macrophages increase Cox-2 mRNA and protein levels; similar inductions were observed when wild-type fibroblasts were incubated with interleukin-β (Zhu et al., 2016). Interestingly, these responses were abrogated in macrophages and fibroblasts derived from Dgkε-null mice. Another line of evidence comes from experiments with wild-type mice treated with the nephrotoxin puromycin aminonucleoside: animals invariably develop glomerular lesions that lead to proteinuria. This phenomenon was shown to be accompanied by robust inductions of Cox-2 expression and urinary prostaglandin excretion (Zhu et al., 2016). Application of the same protocol to Dgkε-null mice revealed relative protection against the proteinuric effects of this toxin, and this effect was correlated with blunted Cox-2 and prostaglandin responses (Zhu et al., 2016). How DGKε activity directly modulates Cox-2 expression has yet to be established. The relevance of these findings to patients with mutations in DGKε is unclear.

The renal phenotypes induced by the subclinical doses of nephrotoxic serum or puromycin are now the main distinctive features of the Dgkε-null mouse model when compared to control littermates. It is unclear if other organs are affected because the bulk of these new investigations were focused on the kidney. While the other Dgk-null animals have no obvious renal phenotypes at baseline [Dgkα (Olenchock et al., 2006), Dgkβ (Shirai et al., 2010), Dgkδ (Crotty et al., 2006), Dgkζ (Zhong et al., 2003), Dgkι (Regier et al., 2005), Dgkη (Isozaki et al., 2016), Dgkθ (Goldschmidt et al., 2016)] it is important to realize that none of these models were exposed to these nephrotoxins. It is therefore not possible to conclude that the renal lesions observed in treated Dgkε–null mice are specific to this animal model.

### Cardiac hypertrophy and heart failure

DGKε is one of the main DGK isoforms expressed in cardiac ventricles; others are DGKα and DGKζ (Takeda et al., 2001). This suggests that DGKε may play an important role in the heart. Decreased DgkE mRNA levels were observed in the hearts of rats used for modeling left ventricular hypertrophy (Yahagi et al., 2005) and myocardial infarction (Takeda et al., 2001). These results did not tease out if this reduction was a normal compensatory mechanism, or if it was an integral part of the disease processes. To start addressing this question, transgenic mice overexpressing DGKε only in the heart were generated (Niizeki et al., 2008). When subjected to two distinct protocols known to induce left ventricular hypertrophy in wild type mice, DGKε-overexpressing mice appeared to be protected (Niizeki et al., 2008). These findings translated into a substantial survival advantage: 4 weeks after the procedure, nearly 80% of DGKε-overexpressing mice were still alive, almost double that of wild type controls (Niizeki et al., 2008). Investigations of well-established biological markers of cardiac pathology corroborate these findings. Upregulation of transient receptor potential channel-6 (Kuwahara et al., 2006) and increased PKCε and PKCα translocation (Hahn et al., 2003; Song et al., 2015) were only observed in wild type mice (Niizeki et al., 2008). Taken together, these results suggest that increasing DGKε function may be a promising target to help prevent heart failure and restore cardiac function. The first step in that direction will be to determine how relevant these data are to patients with cardiac dysfunction. If substantiated, the path to DGKε-based therapy will not be straightforward since it would require tissue-specific overexpression of DGKε.

### Epilepsy and seizure susceptibility

High expression of DGKε in brain tissue suggests that it may play an important role in this organ system (other DGKs are also high in the brain, including β, γ, and ζ) (Zhang et al., 2012). DgkE^−/−^ mice exhibit higher resistance to electroconvulsive shock (ECS) when compared to control littermates (Rodriguez de Turco et al., 2001). A role for DGKε in this process was supported by phosphoinositide quantifications: while wild type mice displayed increased ECS-induced polyphosphoinositide (PIPn) degradation and 20:4 DAG formation, DgkE^−/−^ mice did not (Rodriguez de Turco et al., 2001).

In a similar study, DgkE^−/−^ mice displayed fewer motor seizures, fewer epileptic events, and rapid behavioral recovery following brain stimulation compared to wild type mice (Musto and Bazan, 2006). In addition, wild type mice serially exposed to multiple small seizure events (kindling) developed typical brain morphological changes associated with seizures, but these were absent in DgkE^−/−^ mice (Musto and Bazan, 2006). Kindling induced upregulation of cyclooxygenase-2 (COX-2) and tyrosine hydroxylase (TH) gene expression in wild type mice but not in DgkE^−/−^ mice (Lukiw et al., 2005). High COX-2 expression has been associated with recurrence of hippocampal seizures (Takemiya et al., 2003), and repeated seizures lead to increased TH levels in the brain (Ryu et al., 2000). These data thus suggest that DGKε regulates seizure susceptibility via modulation of COX-2 and TH levels in the brain. It is important to acknowledge that the relevance of these data to humans is unclear as there are no data linking aberrant DGKε function to patients with neurological conditions.

### Huntington's disease

DGKε has been identified as a promising target for treating Huntington's Disease (HD) (Zhang et al., 2012). This condition is characterized by polyglutamine expansion in the N-terminus of the Huntingtin protein (Htt) that causes significant neuronal loss in the striatum and cortex (MacDonald et al., 1993; Zhang et al., 2012). DGK inhibitor II (R59022) was identified as a promising anti-HD compound in a kinase inhibitor library screen. This *in vitro* assay was performed on mouse HD striatal cell model and the readout was the level of mutant Htt cellular toxicity (Zhang et al., 2012). More specifically, R59022 inhibited the expected increase in caspase 3 and 7 activity triggered by serum withdrawal (Zhang et al., 2012). Cells expressing mutant Htt were also found to have lower levels of PIP_*n*_, that can be restored upon decreasing DGK activity (Zhang et al., 2012).

Pinpointing which of the 10 mammalian DGK isoforms was involved in this process required further testing since this compound is a non-specific DGK inhibitor (Sato et al., 2013). siRNA knockdown experiments carried out against the four DGK isoforms expressed in the mouse striatum showed that only DgkE siRNA caused a decrease in caspase 3 and 7 similar to that observed with R59022 (Zhang et al., 2012). Data from two well-established *in vivo* models support the hypothesis suggesting that *in vivo*, enhanced DGKε activity plays a role in HD pathogenesis. First, expression of DgkE shRNA in a *Drosophila* HD model partially rescued the motor impairment induced by Htt (Zhang et al., 2012). Second, DgkE mRNA levels were higher in the striatum of a mouse model of HD (Htt overexpression) (Zhang et al., 2012).

## Future perspectives

The unique primary structure of the smallest known isoform of mammalian DGK, as well as the specificity of DGKε for arachidonoyl-containing DAG, has been known for some time. However, recent findings have presented the possibility of further advances of knowledge in the near future. Two developments have been principally responsible for this. One is the purification of DGKε that will allow new protein structural and membrane binding studies that have not been previously possible. There has also recently been described a causal link between recessive DGKE mutations and a human disease, atypical hemolytic-uremic syndrome (aHUS) (Lemaire et al., 2013). This has given increased importance to defining the biological roles of DGKε in the normal and diseased state.

### Crystal structure

Up to this time, no isoform of a mammalian DGK has been crystallized and its structure determined. However, the crystal structure of a bacterial dgkB from *Staphylococcus aureus*, has been determined (Miller et al., 2008). The amino acid sequence shows 18% identity, with the active site being particularly well conserved (Jennings et al., 2015). A tentative model for the folding of DGKε has been made using the crystallographic structure of dgkB (Jennings et al., 2015).

### Lipid dependence of membrane binding

Binding studies of DGKε with liposomes of defined lipid composition have not yet been carried out because the enzyme was not available in pure form but only as membrane pellets from over-expression systems. Now that we have purified DGKε in solution we can perform such experiments. Dr. Prasanta Hota has done some initial studies in Dr. Epand's laboratory using PIP strips (Echelon Biosciences Inc., Salt Lake City, Utah). It was found that the binding to PI, phosphatidylcholine and lysophosphatidylcholine was very weak, binding to PA, phosphatidylserine, and phosphatidylethanolamine was intermediate and binding to PI with one, two, or three phosphate groups added to the inositol ring was very strong. It is known that phosphorylated forms of phosphatidylinositol are good inhibitors of DGKε (Walsh et al., 1995). Phosphatidylinositol-(4,5)-bisphosphate is a noncompetitive inhibitor with respect to SAG but a competitive inhibitor with respect to ATP (Walsh et al., 1995). This is not purely an electrostatic effect since phosphatidylinositol-trisphosphate is a weaker inhibitor of DGKε than is the diphosphate (Walsh et al., 1995). There is thus likely to be some specificity in the binding of DGKε to membranes containing anionic lipids. The effect of anionic lipids on DGKε binding to membranes has not been fully assessed.

### Activity and substrate specificity in membrane bilayers

There is no reported data on the activity of DGKε using an assay system with phospholipid bilayers. All the reported enzyme activity studies with DGKε have been done with a detergent-solubilized system. Phospholipids in liposomes are arranged as bilayers, which more closely simulates their arrangement in biological membranes. Even the fundamental property of arachidonoyl substrate specificity has not been tested in a bilayer-based system. It also is not clear how membrane binding relates to enzymatic activity of DGKε. It is clear that the DAG substrate is part of the membrane and that DGKε has to bind to a membrane, at least transiently, for phosphorylation to occur. However, as indicated by our preliminary data, anionic lipids strengthen the binding of DGKε to the membrane. Why then don't anionic lipids promote the activity of DGKε as they do for other isoforms of mammalian DGK?

### Development of a DGKε-specific inhibitor

In order to gain a better understanding of DGKε-related diseases, isoform-specific inhibitors should be identified and used in experimental work to delineate the role of DGKε in various tissues and cell types. Although there are several commercially available DGK inhibitors, such as R59022 (de Chaffoy de Courcelles et al., 1985) and R59949 (Sato et al., 2013), there are currently no isoform-specific DGKε inhibitors.

Traditionally, the activities of DGK isoforms were assessed using a micelle-based assay, which utilizes detergents to solubilize lipid components from a lipid film (Epand and Topham, 2007). The hydrated lipid film is then sonicated to produce small unilamellar vesicles. The vesicles are used in a radioactive assay to evaluate the activity of the enzyme, by measuring the transfer of ^32^P from [γ-^32^P]-ATP to DAG to form PA (Epand and Topham, 2007). Although this procedure is quite insensitive to non-DGK ATPase activity, the assay might not be well suited for a high-throughput system due to the extensive radioactive and extraction procedures.

Recently, an ATP-luciferase system was developed to evaluate the isoform selectivity of the R59949 and R59022 inhibitors for various DGKs (Sato et al., 2013). The assay was later optimized to study DGKα, and was used to identify a novel DGKα-specific inhibitor, CU-3 (Liu et al., 2016). Similarly, the ATP-luciferase assay can be optimized to study DGKε in a high-throughput format to identify a DGKε-specific inhibitor. Due to the fact that DGKε utilizes ATP as a phosphate donor in the conversion of DAG into PA, the concentration of ADP generated can be used as a measure of PA production and enzyme activity (Shulga et al., 2011c). Since the ATP-luciferase assay detects the ADP released in the kinase reaction, the assay must utilize purified DGKε, in order to reduce contamination with other sources of ATPase activity (Zegzouti et al., 2009).

Once a pool of candidate inhibitors has been identified using the high-throughput method, the candidates can be validated using various assays. For example, the traditional micelle-based radioactive assay or the liposome based assay currently being developed can be used to confirm changes in enzyme activity in the presence of inhibitors (Epand and Topham, 2007). In addition, a ^31^P nuclear magnetic resonance assay (NMR) can be used to identify phosphorous containing substrates and products and detect potential contaminants with ATPase activity (Prodeus et al., 2013). Lastly, the ATP-luciferase assay can be optimized to assess the activity of various other DGK isoforms. Once optimized, the assay would be used to the test the inhibitory potential of candidate compounds against various DGK isoforms, to assess the isoform specificity of novel inhibitors.

The development of a DGKε-specific inhibitor would be invaluable for studying the role of DGKε in various disease processes. Specifically, the isoform-specific inhibitor could be used to recapitulate physiological environments lacking DGKε. In addition, the luciferase assay holds potential as a diagnostic tool for measuring the enzyme activity of DGKε in patients (or with CRISPR-Cas9 mutated cells) with missense DGKε mutations.

### Possible modulation of DGKε's transmembrane vs. re-entrant helix

The amino terminal hydrophobic segment of DGKε is highly conserved in evolution (Figure 3). In particular, there is an invariant proline residue at position 33 in human DGKε that is present at the same position of the aligned sequences from all DGKε from a range of organisms (Supplementary Materials). We have shown that this proline residue has an important role in determining the position of equilibrium between a transmembrane helix and a re-entrant helix (Decaffmeyer et al., 2008). This segment of the protein has no effect on the activity or specificity of DGKε using an *in vitro* assay in detergent micelles with N-terminal 40–60 residue deletion mutants. However, we believe that it is unlikely that this segment of the protein would not have a biological function. Forming a re-entrant helix would promote the positive curvature of the monolayer in which it is present. This could influence the region of the membrane to which DGKε partitions and/or modulate its interaction with other proteins. An understanding of the role of this proline residue and possibly of the interconversion between re-entrant and transmembrane helix is a theme for future investigations.

## Author contributions

All authors listed, have made substantial, direct and intellectual contribution to the work, and approved it for publication.

## Funding

Supported by a grant from the Natural Sciences and Engineering Research Council of Canada (Grant 9848, to RE).

### Conflict of interest statement

The authors declare that the research was conducted in the absence of any commercial or financial relationships that could be construed as a potential conflict of interest.

## Abbreviations

aHUS: atypical hemolytic-uremic syndrome
arrb: arrestinβ
CDS: CDP-DAG synthase (Phosphatidatecytidylyltransferase)
DGK: diacylglycerol kinase
DAG: diacylglycerol
ECS: electroconvulsive shock
ER: endoplasmic reticulum
GK: glycerol kinase
GPCR: G-protein coupled receptor
HD: Huntington's disease
Htt: Huntington protein
MEFs: mouse embryo fibroblasts
NeoR: neomycin resistant
PA: phosphatidic acid
PI: phosphatidylinositol
PI-cycle: phosphatidylinositol-cycle
PIP2: phosphatidylinositol-(4, 5)-bisphosphate
PIP_*n*_: phosphorylated forms of PI
SAG: 1-stearoyl-2-arachidonoyl glycerol
TH: tyrosine hydroxylase.
