# Supplementary material for: Diacylglycerol Kinase-ε: Properties and Biological Roles
Source: Front Cell Dev Biol. 2016 Oct 18;4:112. doi: 10.3389/fcell.2016.00112 (PMC5067486; doi:10.3389/fcell.2016.00112)
Supplement: Supplementary file 1 [file Table1.pdf]

Supplemental Figure 1

Excerpts from DGKε sequence alignment from different Vertebrate and some invertebrate species showing the conservation of Proline residue at position 33 (highlighted) in the sequence.

Group	Species	ID	25–41
Primates	Homo sapiens	5616188	WTLCSVLLPVFITFWCS
	Gorilla gorilla gorilla	426347426	WTLCSVLLPVFITFWCS
	Pan troglodytes	694968683	WTLCSVLLPVFITFWCS
	Pan paniscus	675742782	WTLCSVLLPVFITFWCS
	Pongo abelii	686753387	WTLCSVLLPXVITFWCS
	Macaca fascicularis	67972066	WTLCSVLLPVFITFWCS
	Macaca mulatta	297272634	WTLCSVLLPVFITFWCS
	Macaca nemestrina	795620848	WTLCSVLLPVFITFWCS
	Chlorocebus sabaeus	635089833	WTLCSVLLPVFITFWCS
	Cercocebus atys	795571756	WTLCSVLLPVFITFWCS
	Rhinopithecus roxellana	724909225	WTLCSVLLPVFITFWCS
	Colobus angolensis palliatus	795243343	WTLCSVLLPVFITFWCS
	Papio anubis	685597505	WTLCSVLLPVFITFWCS
	Callithrix jacchus	296202403	WTLCSVLLPVFITFWCS
	Aotus nancymaae	817291140	WTLCSVLLPVFITFWCS
	Saimiri boliviensis boliviensi	403279700	WTLCSVLLPVFITFWCS
	Otolemur garnettii	395845794	WTLCSVLLPVFITFWCS
	Tarsius syrichta	640826139	WTLCSVLLPVFITCWCS
	Propithecus coquereli	826274413	WTLCSVLLPVFITFWCS
	Microcebus murinus	29719269	WTLCSVLLPVFITFWCS
Rodents	Mesocricetus auratus	24951727	WTLCSVLLPVFITFWCS
	Nannospalax galili	74060070	WTLCSVLLPVFITFWCS
	Cricetulus griseus	44243468	WTLCSVLLPVFITFWCS
	Peromyscus maniculatus bairdii	89917547	WTLCSVLLPVLITLWCS
	Cricetulus griseus	25188058	WTLCSVLLPVFITFWCS
	Mus musculus	6336623	WTLCSVLLPVFITLWCS
	Microtus ochrogaster	32013590	WTLFSVLLPVFITFWCS
	Rattus norvegicus	49053838	WTLFSVLLPVFITLWCS
Other Mammals	Chrysochloris asiatica	86449525	WTLCSVLLPVFITFWCS
	Elephantulus edwardii	85671592	WTLCSVLLPVFITFWCS
	Dasypus novemcinctus	21140633	WTLCSVLLPVFITFWCS
	Tupaia chinensis	47277591	WTLCSVLLPVFVTFWCS
	Loxodonta africana	44285375	WTLCSVLLPVFITFWCS
	Orycteropus afer afer	34872742	WTLCSVLLPVFITFWCS
	Trichechus manatus latirostris	71380322	WTLCSVLLPVFITFWCS
	Monodelphis domestica	11990756	WTLCSVLLPVLITLWCS
	Sarcophilus harrisii	21480855	WTLCSVLVPVLITLWCN
	Ornithorhynchus anatinus	20985162	WTLCSVLLPVLLTLWCS
	Equus caballus	545179273	WTLCSVLLPVFITFWCS
	Chinchilla lanigera	533160536	WTLCSVLLPVFITFWCS
	Heterocephalus glaber	512996527	WTLCSVLLPVFITFWCS
	Fukomys damarensis	676281227	WTLCSVLLPVFITFWCS
	Dipodomys ordii	852732227	WTLCSVLLPVFITFWCS
	Leptonychotes weddellii	585194198	WTLCSVLLPVFITFWCS
	Ailuropoda melanoleuca	301759659	WTLCSVLLPVFITFWCS
	Mustela putorius furo	511846013	WTLCSVLLPVFITFWCS
	Octodon degus	507704592	WTLCSVLLPVFITFWCS
	Ceratotherium simum simum	478521197	WTLCSVLLPVFITFWCS
	Panthera tigris altaica	591321210	WTLCSVLLPVFITFWCS
	Felis catus	410980673	WTLCSVLLPVFITFWCS
	Canis lupus familiaris	73966623	WTLCSVLLPVFITFWCS
	Ovis aries	803112718	WTLCSVLLPVFITFWCS
	Capra hircus	926715761	WTLCSVLLPVFITFWCS
	Bubalus bubalis	594106732	WTLCSVLLPVFITFWCS
	Bos taurus	300793691	WTLCSVLLPVFFTFWCS
	Bison bison bison	742175154	WTLCSVLLPVFFTFWCS
	Bos mutus	555987838	WTLCSVLLPVFFTFWCS
	Camelus ferus	560910323	WTLCSVLLPVFITFWCS
	Vicugna pacos	560988630	WTLCSVLLPVFITFWCS
	Physeter catodon	593727630	WTLCSVLLPVFITFWCS
	Orcinus orca	466019059	WTLCSVLLPVFITFWCS

Other Mammals	Lipotes vexillifer	602707331	WTLCSVLLPVFITFWCS
	Balaenoptera acutorostrata sca	594655682	WTLCSVLLPVFITFWCS
	Pteropus alecto	586540170	WTLCSVLLPVFITFWCS
	Cavia porcellus	348562564	WTLCSVLLPVFITFWCS
	Ursus maritimus	671005957	WTLCSVLLPVFITFWCR
	Sorex araneus	505802581	WTLCSVLLPVFITFWCS
	Sus scrofa	264681444	YTLCSVLLPVFITFWCS
	Myotis brandtii	554556336	WTLCSVLLPVFITFWCS
	Eptesicus fuscus	641713396	WTLCSVLLPVFITFWCS
	Jaculus jaculus	507572892	WTLCSVLLPVFITLWCS
	Erinaceus europaeus	617618893	WTLCSVLLPVLITCWCS
	Oryctolagus cuniculus	291405752	WTLCSVLLPVAVTLWCS
	Ochotona princeps	504168622	WTLCSVLLPVAVTLWCS
	Alligator sinensis	557263924	WTLCSVVVPVLITLWCS
	Alligator mississippiensis	950994220	WTLCSVVVPVLITLWCS
	Chelonia mydas	591360518	WTLCSVILPVVITLWCS
	Chrysemys picta bellii	641760021	WTLCSVILPVVITLWCS
	Condylura cristata	830038813	WTLCSVLLPVFITFWCS
	Chaetura pelagica	701422173	WTLCSVLLPVLITLWCS
Reptiles	Calypte anna	663263247	WTLCSVLLPVLITLWCS
	Opisthocomus hoazin	700392595	WTLCAVLLPVLITLWCS
	Picoides pubescens	678196712	WTLCSVLLPVLITLWCS
	Serinus canaria	683924150	WTLCSVLLPVLITAWCS
Birds	Tauraco erythrolophus	701327655	WTLCAIILPVLITVWCS
	Anser cygnoides domesticus	902889883	WTLCSVIVPVLITLWCS
	Balearica regulorum gibbericep	723547949	WTLCSVLLPVLITLWCS
	Gavia stellata	698449606	WTLCSVILPVLITLWCS
	Amazona aestiva	944212817	WTLCSVLLPVLITVWCS
	Nipponia nippon	694848337	WTLCSVILPVLITLWCS
	Columba livia	543723927	WTLCAVILPVLITLWCS
	Aptenodytes forsteri	686617944	WTLCSVILPVLITLWCS
	Haliaeetus leucocephalus	729760143	WTLCSVILPVLITLWCS
	Phalacrocorax carbo	XP_009505860	WTLCSVLLPVLITLWCS
	Chaetura pelagica	XP_010000988	WTLCSVLLPVLITLWCS
	Gavia stellata	XP_009818613	WTLCSVILPVLITLWCS
	Aquila chrysaetos canadensis	XP_011585227	WTLCSVILPVLITLWCS
Fishes	Pygocentrus nattereri	XP_017548559	WTGLAVLLPILITLWCS
	Sinocyclocheilus rhinocerous	XP_016370480	WTTVAVLVPVLITLWCS
	Astyanax mexicanus	XP_007234459	WTSLAILLPVLITLWCS
	Danio rerio	XP_001920687	WTTFAVLVPVLITLWCS
	Ictalurus punctatus	XP_017337227	WTGLAVLLPVLITLWCS
Arthropoda	Papilio polytes	XP_013141971	CQLCDTYFPVYQMEWCS
	Papilio xuthus	XP_013167633	CQLCDTYFPVYQMEWCS
	Papilio machaon	XP_014363046	CQLCDTYFPVYQMEWCS
